# Tough Structural Adhesives with Ultra-Resistance to Both High and Cryogenic Temperature

**DOI:** 10.3390/polym15102284

**Published:** 2023-05-12

**Authors:** Hui Niu, Shengtao Wang, Yilin Shen, Shouqing Liu, Shuyang Jiang, Tao Qin, Taohong Li

**Affiliations:** 1 The Yunnan Provincial Key Laboratory of Wood Adhesives and Glued Products, Southwest Forestry University, Kunming 650224, China; m18841088597@163.com (H.N.); stwang1029@163.com (S.W.); shenyilin1118@163.com (Y.S.); jsy_poxxyy@163.com (S.J.); wsyjs910@163.com (T.Q.); 2 College of Material Science and Chemical Engineering, Southwest Forestry University, Kunming 650224, China; 3 International Joint Research Center for Biomass Materials, Southwest Forestry University, Kunming 650224, China

**Keywords:** structural adhesive, melamine, M–Xylylenediamine, bonding strength, high-temperature resistance

## Abstract

Structural adhesion at high temperature has been a challenge for organic adhesives, and the commercially available adhesives that can work at a temperature above 150 °C is rather limited. Herein, two novel polymers were designed and synthesized via facile strategy, which involves polymerization between melamine (M) and M–Xylylenediamine (X), as well as copolymerization of MX and urea (U). With well-balanced rigid-flexible structures, the obtained MX and MXU resins were proved to be outstanding structural adhesives at a wide range temperature of −196~200 °C. They provided room-temperature bonding strength of 13~27 MPa for various substrates, steel bonding strength of 17~18 MPa at cryogenic temperature (−196 °C), and 15~17 MPa at 150 °C. Remarkably, high bonding strength of 10~11 MPa was retained even at 200 °C. Such superior performances were attributed to a high content of aromatic units, which leads to high glass transition temperature (T_g_) up to ~179 °C, as well as the structural flexibility endowed by the dispersed rotatable methylene linkages.

## 1. Introduction

High-performance polymers play important roles in modern industrial systems covering civil and high-tech fields, including building, vehicle, electronics, military, aerospace, energy, etc., where the polymers are used as adhesives, composite matrices, sealants, coatings, etc. [1,2,3]. For structural use, the polymers are required to bear excellent mechanical properties and durability, especially outstanding resistance to high and cryogenic temperatures. High heat resistance is dependent on the high thermostability of the polymer structure, which is described by decomposition temperature (T_d_) and glass transition temperature (T_g_). Differently, durability to cryogenic temperature is closely related to toughness, which is determined by the structural flexibility at low temperatures [4,5,6]. To achieve these desired properties, careful structure design and synthesis are necessary.

The structural adhesive is one of the engineering applications of high-performance polymers. Currently, the conventional mainly include phenolic [7], epoxy [8], acrylic [9], cyanate [10], and polyurethane [11]. However, commercial adhesives that can provide long-term service at temperatures above 150 °C are very limited, and the adhesives that can work at 200 °C are rare. Among these adhesives, epoxy has been developed as the largest family and is currently the most used resin due to its easy application and excellent room temperature adhesion property for diverse substrates. By modifications or reinforcement, better high-temperature adhesion strength can be achieved. For example, the thermoplastic block copolymer (TPBC) [12] and polyphenoxy (PHO) and boron nitride (BN) [13] modified epoxy resins exhibited satisfied high-temperature bonding performance at 120 and 150 °C. Polyurethane-based adhesives possess the best cryogenic adhesion property, but they generally give poor adhesion strength at high temperatures [14,15]. In addition, commercial polyurethane adhesives usually contain free N=C=O groups that are highly reactive to water, so the cure of the adhesives is affected by even a small amount of moisture [16]. Moreover, polyurethane adhesives represent healthy risks if not handled properly. Polyimide and bis-maleimide adhesives are the representatives of high-temperature polymers as they have extremely high T_g_ (280~400 °C) and decomposition temperature (T_5%_ = 400~500 °C) [6,17,18,19]. However, their extremely high price and poor processibility largely restricted their applications.

**Scheme 1:**
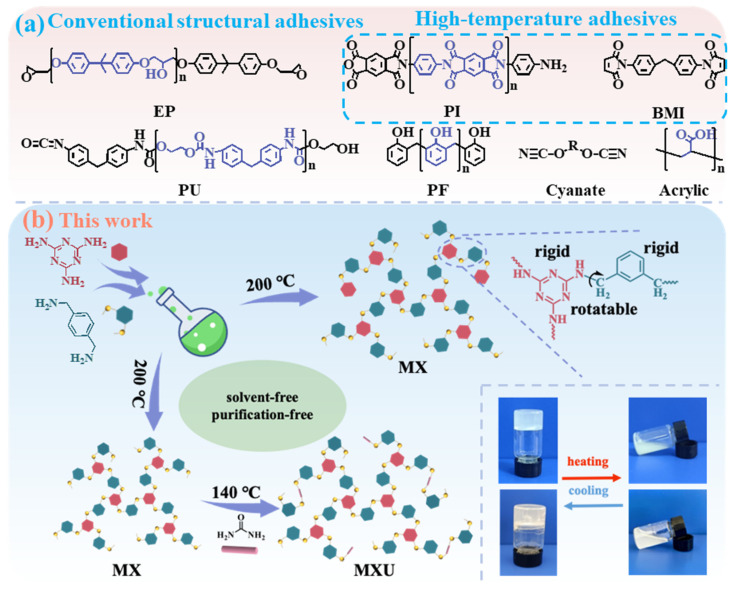
(**a**) Chemical structures for conventional structural and high-temperature adhesives (Key: EP = epoxy resin, PU = polyurethane, PF = phenolic resin, PI = Polyimide, BMI = bis-maleimide). (**b**) Synthetic routes, structures, and appearance of MX and MXU resins in this work.

In contrast to thermoplastic resins, thermosetting resins can generally provide higher mechanical strength and thermostability due to crosslinked structure [20], but meanwhile, the flexibility is sacrificed. Theoretically, the incorporation of both flexible and rigid fragments into molecular chains could lead to high strength and toughness. However, this is determined by what monomers are used and how the structure is balanced. In fact, designing a polymer that can resist both high and cryogenic temperatures is usually a great challenge. According to the designing strategy for high-performance polymers such as polyimide and bis-maleimide (Scheme 1a), rich aromatic fragments and a highly conjugated backbone (resonance stabilization) are important for thermostability [21,22,23]. However, a highly rigid structure inevitably increases brittleness. Recently, we developed a novel thermoset synthesized using melamine (M) and 1,6-hexamethylenediamine (H) as monomers [24]. Thanks to the stable covalent bonds and rigid plus flexible structure, the obtained MH resin exhibited high decomposition temperature (T_5%_ ≈ 460 °C) and excellent mechanical properties (strong and tough) at both ambient and cryogenic temperatures (−196 °C). However, due to its highly flexible structure, the T_g_ of the resin is relatively low (≈90 °C, determined by DSC). Therefore, the high-temperature performance of the resin is not satisfactory. To obtain well-balanced rigidity and flexibility, in this work, we re-designed the polymer structure by alternating 1,6-hexamethylenediamine with M–Xylylenediamine (X). The formulation of the resulting MX polymer is totally different from the conventional structural adhesives and high-temperature adhesives (Scheme 1a). With rich aromatic units, the polymer became more rigid than MH and exhibited much higher T_g_. With the presence of a rotatable sp^3^ carbon (methylene group), the molecular chains were endowed with a certain flexibility so that the cured resin could also resist cryogenic temperature. Particularly, the MX resin exhibited excellent adhesion properties for various substrates at a temperature range of −196~200 °C. By incorporating urea (U) as the third monomer, the MXU resin was also synthesized. This resin is less thermally stable than MX but demonstrated lower cure temperature and higher bonding strength.

## 2. Experimental Section

### 2.1. Materials

Melamine (M, analytical reagent, AR), Ammonium Chloride (NH_4_Cl, analytical reagent, AR), and Urea (U, analytical reagent, AR) were purchased from Sinopharm Chemical Reagent Co., Ltd. M–Xylylenediamine (X, analytical reagent, AR) were purchased from Shanghai Macklin Biochemical Co., Ltd. Ceramic, aluminum (Al), stainless steel, and copper (Cu) sheets, as well as composite carbon fiber panels (CF) were purchased from a local store.

### 2.2. Synthesis of MX Resins

In the synthesis of MX resin, X, M, and the catalyst NH_4_Cl (6% of the weight of M) were mixed under stirring in a round-bottom flask. Then the mixture was steadily heated to 200 °C in an oil bath. The released ammonia gas was absorbed using a water trap. When a fast increase in viscosity was observed, the mixture was immediately poured out and cooled to room temperature. With this method, syntheses with a wide range M:X ratio of 1.0:1.0~3.0 were performed. However, when a 1.0:1.0 molar ratio was used, the mixture lost flowability within 1 h, which could lead to a low polymerization degree. When a molar ratio of 1.0:3.0 was applied, the obtained polymer was incurable. Finally, three M:X molar ratios of 1.0:1.5, 1.0:2.0, and 1.0:2.5 were identified as optimal ones. With these molar ratios, a fast increase of viscosity occurred at 3~4 h, and the cooled resins appeared to be white solid. The obtained resins were marked as MX-1.5, MX-2.0, and MX-2.5, respectively.

### 2.3. Synthesis of MXU Resins

The synthesis of MXU resins was divided into two steps. First, X, M, and the catalyst NH_4_Cl (6% of the weight of M) were mixed under stirring in a round-bottom flask. Then the mixture was steadily heated to 200 °C in an oil bath. The released ammonia gas was absorbed using a water trap. After M was completely dissolved, the mixture was cooled to 140 °C, and U was added. When the mixture became light yellow and transparent liquid, as well as a fast increase of viscosity was observed, the reaction product was immediately poured out and cooled to room temperature. With this procedure, Several MXU resins were synthesized with different M:X:U molar ratios of 1:3:2, 1:3:3, 1:3:4, 1:4:2, 1:4:3, and 1:4:4 were synthesized, and they were marked as MXU-132, MXU-133, MXU-134, MXU-142, MXU-143, and MXU-144, respectively.

### 2.4. Electrospray Ionization Mass Spectrometry (ESI-MS) Characterization

Prior to analysis, MX or MXU resin was dissolved in ethanol–water mixtures (70% [*v*/*v*]) with a concentration of 1 mg·mL^−1^. Mass spectrometric analysis was performed on a quadrupole time-of-flight (Q-TOF) high-resolution mass spectrometer (Q-TOF liquid chromatography/mass spectrometry (LC/MS) 6540 series, Agilent Technologies, Santa Clara, CA) coupled with electrospray ionization (ESI). The detection was performed in a positive ESI mode.

### 2.5. Gel Permeation Chromatography (GPC)

GPC was carried out on a Waters1525 GPC system equipped with a PLgel Olexis 300 × 7.5 mm gel column, and the column’s temperature was maintained at 35 °C. Detection was performed on a 2414 refractometer using DMSO as the eluent at a flow rate of 1 mL·min^−1^. The calibration curve, determined by a series of narrowly distributed glucan standards, was employed to calculate the relative molar masses of the samples.

### 2.6. ^13^C Nuclear Magnetic Resonance (NMR) Characterizations

The ^13^C nuclear magnetic resonance (^13^C-NMR) elucidations were performed using a Bruker AVANCE III 500 spectrometer (Bruker Corporation, Billerica, MA, USA). A total of 20 mg of solid sample was directly dissolved in 500 μL of DMSO-d6 for ^13^C-NMR analysis. The observed chemical shifts were assigned by referring to the spectra of pure M–Xylylenediamine.

### 2.7. Thermogravimetric Analysis (TGA)

To investigate the cure behavior and thermostability of MX and MXU resins, TGA runs for un-cured and cured resins were performed on a thermogravimetric analysis instrument (NETZSCH TG 209 F3, Selb, Germany) over the temperature range from 30 to 800 °C in N_2_ atmosphere at a heating rate of 10 K/min.

### 2.8. Differential Scanning Calorimetry (DSC) Analysis

The resin samples (5~10 mg) cured at 250 °C for 30 min were placed in aluminum DSC pans and tested using a DSC 204 F1 (NETZSCH, Selb, Germany) in a temperature range of 30~300 °C at a heating rate of 10 °C/min under N_2_ atmosphere.

### 2.9. Tests of Bonding Strength

In the preparation of specimens, metal substrates, including stainless steel, copper, Al, carbon fiber composite, and ceramic sheets, were sanded to remove possible surface oxidants oxides or other plating layers (or film). Then the sanded surface was washed with water, ethanol, and acetone and dried in an oven at 100 °C for 20 min. Finally, the melted MX or MXU resin was spread on the substrate surface in an area of 25 mm × 10 mm (20 × 10 mm for ceramic sheet). Then two sheets were overlapped and fastened with a clip. Before tests for all substrates, specimens prepared with steel were used to optimize curing conditions, including cure temperature (210~270 °C) and time (30~120 min). With the optimized conditions, tests for other substrates were carried out.

By referring to the standard of GB/T 7124-2008, lap shear bonding strength tests were conducted on a universal mechanic machine (Dongguan Huakai Testing Equipment Technology Co., Ltd., Dongguan, China). For room temperature bonding strength, tests were carried out at ambient air temperature and humidity. For the tests at cryogenic temperature (liquid nitrogen (−196 °C)) and high temperature (80~200 °C), two self-designed devices were used with a universal mechanic machine. Specifically, to create a cryogenic environment, a device that combined a specimen and a container (plastic cup) was designed. In the test, liquid nitrogen was continuously poured into the container to ensure the overlapped area of the specimen was soaked in liquid nitrogen during the testing process. For tests at high temperatures, the specimen was packed in a self-designed electric heating jacket. For each test, the specimen was heated to the desired temperature and kept for 10 min. The set temperature was maintained during the testing process. For a better understanding of the details of the two devices, a figure was drawn and can be seen in the results and discussion section. For all the tests of lap shear strength, the tensile speed of 5 mm/min was applied. For each test, the average bonding strength was obtained from five duplications.

### 2.10. Tests of Chemical Resistance

The bonded steel specimens were immersed in 2 mol·L^−1^ NaOH solution and 2 mol·L^−1^ H_2_SO_4_ solution for 72 h, respectively. Then, after the specimens were rinsed several times using deionized water and dried in an oven at 80 °C, the residual lap shear strengths were measured.

## 3. Results and Discussion

**Figure 1:**
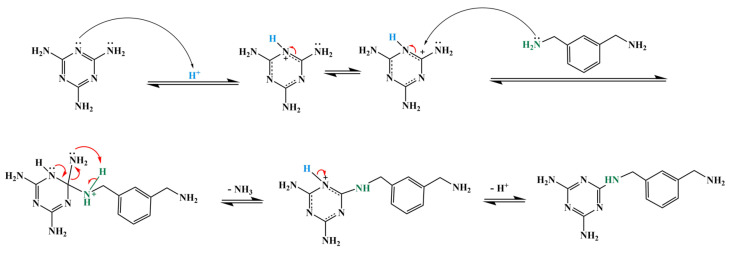
The proposed mechanism for M–X condensation reaction.

Scheme 1b demonstrated the synthetic routes for MX and MXU resins. Similar to MH synthesis, this strategy is also based on the deamination condensation between aliphatic amine and melamine or urea. Figure 1 shows the proposed condensation mechanism for MX synthesis. In such a mechanism, NH_4_Cl plays the role of a proton donor, and the nitrogen in the triazine ring could be protonated due to its relatively strong Lewis alkalinity originating from its lone-paired electron located in one of the sp^2^ orbitals, which does not conjugate with the triazine π bond. Once protonation occurred, the adjacent sp^2^ carbon became highly reactive toward the aliphatic amino group (–NH_2_). The formation of the MXU polymer should also share the same mechanism. Taking the advantages of the low melting point (~14 °C) and high boiling point (~247 °C) of M–Xylylenediamine (X), solvent-free synthesis of MX at 200 °C was carried out to avoid purification after synthesis. Melamine is well known for its poor solubility in various organic solvents, which frequently leads to low efficiency in synthesis. A valuable advantage of X over H is its excellent dissolution capability toward melamine. The previous synthesis of MH resin took 20~30 h (depending on H:M molar ratio) even at 200 °C. One of the important reasons is the low solubility of M in H, resulting in a low-rate heterogeneous reaction. Differently, certain solubility of M in X, even at room temperature, was observed. When the temperature was elevated to 200 °C, M completely dissolved in X within 1~2 h, and the whole synthesis took only 3~4 h. The good solubility of M in X may be ascribed to the strong M–X π–π interaction, which can rapidly break up assembled melamine solids or efficiently destroy the crystal structure of melamine.

To investigate the effect of X:M molar ratios on the properties of final resins, molar ratios of 1.5, 2.0, and 2.5 were used. Statistically, for A_2_ + B_3_ polymerization, a molar ratio of 2.0 can lead to an X-terminated polymer if the reaction equilibrium is established. However, in practice, once the target viscosity is observed, the reaction is stopped. Therefore, even if excessive X is used, polymers containing both terminal M and X can be obtained. Thus, at higher temperatures, further polycondensations can lead to a crosslinked network, resulting in the cured resin. In the synthesis of MXU, MX polymers were synthesized firstly using a higher X:M molar ratio of 4.0 to ensure the majority of the polymers were terminated by X. Considering urea is more reactive than M, in the second step, the temperature was decreased to 140 °C. At this temperature, the reaction is controllable, especially U–U condensation that may cause crosslinking during synthesis can also be avoided. Three MXU resins were synthesized using molar ratios of 1:4:2, 1:4:3, and 1:4:4. Theoretically, the resulting polymers should contain M, X, and U terminals. As shown in Scheme 1b, at room temperature, the uncured MX and MHU resins appear to be white and light-yellow solids, respectively, and they became liquid (melted) when heated to about 120 °C.

**Figure 2:**
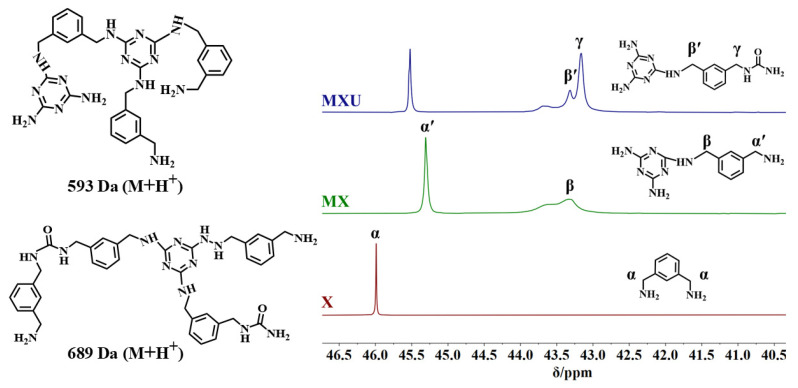
The assigned possible structures for MX and MXU polymers, based on the selected peaks in ESI-MS spectra and the ^13^C-NMR spectrum for pure X, MX-1.5, and MXU-144 resins.

To confirm the structures of MX and MXU polymers, ESI-MS, ^13^C-NMR, and GPC characterizations were performed by selecting MX-1.5 and MXU-144 as representatives. The combined results are given in Figure 2. The original spectra are shown in Figures S1–S9. The assigned structures for the selected ESI-MS peaks suggested that the expected MX and MXU polymers have been successfully formed. The changes of the ^13^C-NMR signals of the sp^3^ methylene carbon further confirmed the structure of the polymers. In contrast to the peak **α** at 46.26 ppm in X, the peak **α′** in MX slightly shifted to a high field and appeared at 45.57 ppm in MX. A similar change can also be seen for peak **α″** (45.80 ppm) in MXU. Therefore, M–X polymerization led to an increased shielding effect on the unreacted methylene carbon, but the effect is very slight. Differently, once the amino hydrogen was substituted by melamine, such a shielding effect was significantly enhanced as the new peak **β** for the methylene carbon shifted significantly to 43.64 ppm in MX, implying the amino triazine plays the role of electron donor to the methylene carbon. This feature further confirmed the formation of M–X linkages. For MXU, the new peak split into two peaks at 43.59 ppm (**β′**) and 43.43 ppm (**γ**), corresponding to M–X and M–U linkages, respectively. The GPC results listed in Table 1 show that the MX resin has M_n_ above 1.5 × 10^4^ and M_w_ above 8.3 × 10^4^, indicating wide range distribution of molecular weight and inevitably leading to a high PDI of 5.45. For a thermoset, the negative effect of high PDI would not be significant as the small and large polymers will condense further to form a crosslinked network during the curing process. Note that M_n_ and M_w_ of previously synthesized MH were around 7 × 10^3^ and 1.4 × 10^4^ [24], respectively, much lower than that of MX resin; even a much longer reaction time of 20~30 h was applied. Such a significant difference cannot be rationalized by the structural difference between H and X, as they are both aliphatic amines, and their nucleophilicity should be similar. The benzyl nature of X should not be the reason because X plays the role of attacking agent, not attacked substrate. As mentioned above, higher solubility of M in X than in H should be responsible for the significantly shortened reaction time and higher polymerization degree.

For MXU-144, the products of the two reaction steps were separately analyzed. The M_n_ and M_w_ of the first-step MX product are ~2.1 × 10^4^ and ~9.3 × 10^4^, respectively. After U was added in the second step at a lower temperature of 140 °C, M_n_ increased to ~2.4 × 10^4^, but M_w_ decreased to ~7.3 × 10^4^. This indicates that the addition of U did not lead to crosslinking between the large polymers. A slight increase of M_n_ suggests that co-condensations between U and MX polymers have occurred, leading to MXU polymers, especially a portion of small MX polymers formed at the first step, becoming bigger at the second step due to the linking of U units. The decrease of M_w_ indicates that the large polymers with the highest molecular weight did not become larger. Thus, the increase in the middle-size polymers inevitably leads to a decreased contribution of the largest polymers to the overall weight.

**Figure 3:**
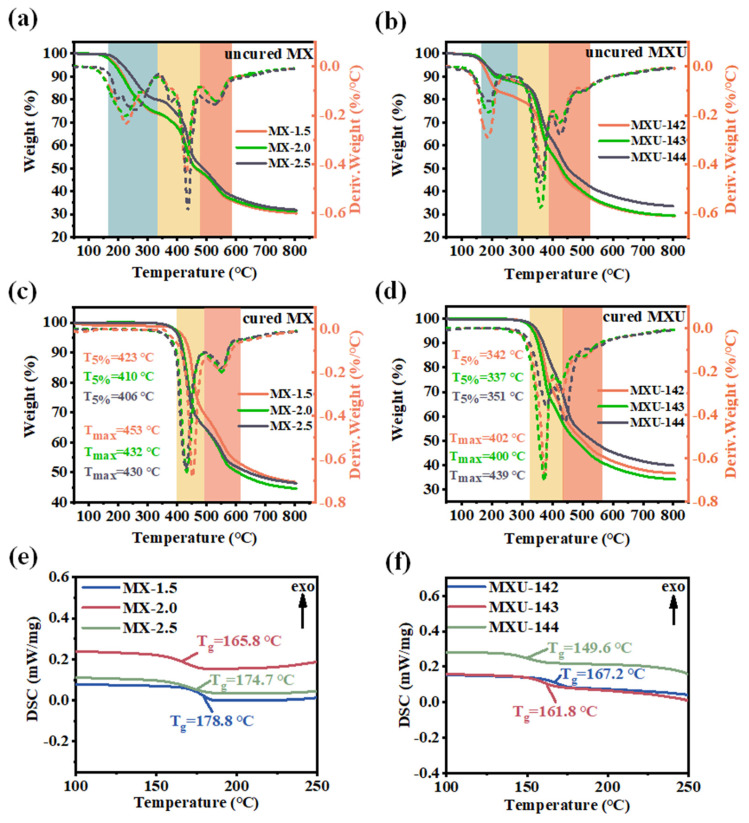
(**a**,**b**) The TGA results for uncured MX and MXU resins. (**c**,**d**) The TGA resiles for cured MX and MXU resins. (**e**,**f**) The DSC results for cured MX and MXU resins.

To investigate the cure behaviors and thermostability of the two resins, TGA analyses were carried out for the uncured resins synthesized with different molar ratios. Weight loss caused by the elimination of NH_3_ can be an indication of the curing process. The weight-loss curve, along with an increase in temperature in Figure 3a, shows a step-wise weight loss for uncured MX resin. The first-stage loss occurred at approximately 230~270 °C, corresponding to an early cure of MX. Post-cure was extended to about 400 °C. This curing process is similar to that of MH, confirming that the reactivity of X is similar to H. Figure 3b shows that the cure of MXU resin occurred approximately at 160~350 °C, corresponding to the formation of X–U, U–U, and M–X linkages. For the cured MX resin, T_5%_ was determined at 406~423 °C (Figure 3c); this is much lower than that of 460 °C of MH resin. The fast decomposition at around 450 °C is likely caused by the breakage of the methylene linkages -M-CH_2_-X-. Then we may speculate that the -M-CH_2_-X- structure is less stable than -M-[CH_2_]_6_-M-. When the cured network is heated, the absorbed energy can be better delocalized by the longer and more flexible chain via stretching deformation (vibration), resulting in delayed decomposition. Therefore, the more rigid structure of MX than MH did not lead to higher decomposition temperatures. Nevertheless, the T_5%_ of MX surpasses most engineering polymers and is close to the general criterion for high-temperature polymers (>450 °C). For the cured MXU resin (Figure 3d), the T_5%_ at around 337~351 °C is related to the decomposition of the -NH-CO-NH- linkages. For both MX and MXU, the high content of aromatic fragments leads to a high char yield of 40~45% at 800 °C, which is similar to phenolic resins.

Figure 3e shows the T_g_ for MX resins synthesized using three molar ratios. An obvious trend is that a higher X:M ratio led to higher T_g_, implying that excess of X may result in decreased crosslinking density as condensation cannot occur between excess X terminals. As expected, the T_g_ of 165~179 °C is much higher than that of MH (~90 °C). Therefore, although the more rigid structure did not lead to higher decomposition temperature, a significant elevation of T_g_ was realized. Further, the T_g_ ≈ 178.8 of MX-1.5 was already close to the criteria for a high-temperature polymer (T_g_ > 200 °C)^21^, implying the resin would exhibit excellent thermal-mechanical properties. In contrast, the T_g_ of MXU resins (Figure 3f) is slightly lower (150~167 °C) than that of MX but much higher than that of MHU (~110 °C). Additionally, the short chain, strong π–π stacking interactions between the aromatic fragments should also be an important effect that contributed to the increased T_g_.

**Figure 4:**
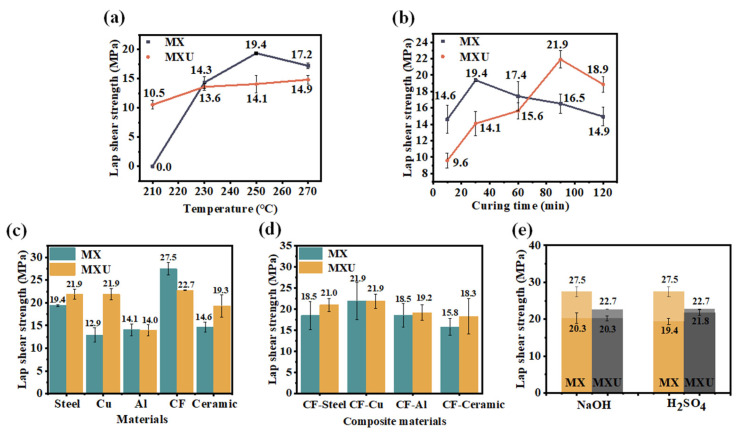
(**a**) Effects of cure temperature on steel bonding strength (30 min curing). (**b**) Effects of curing time on steel bonding strength (250 °C). (**c**) Bonding strengths for different substrate materials. (**d**) Bonding strengths for composite substrate materials. (**e**) Resistance to NaOH and H_2_SO_4_ solutions (2.0 mol/L) by CF bonding strength tests.

In a previous study, MH and MHU resins exhibited excellent adhesion strength for various substrates at room and cryogenic temperatures [24]. However, due to the relatively low T_g_, the bonding strength declined sharply from 20~25 MPa to 2~5 MPa once the ambient temperature was elevated to 140 °C, restricting its application from a high-temperature environment. Since the T_g_ of MX and MXU have been elevated, their bonding performance at different temperatures should be largely improved and demonstrate a significant advantage over MH and MHU resins. Before the resins were applied to different substrates, curing conditions were first optimized via steel bonding tests. In these tests, the MX-1.5 and MXU-144 resins were also selected as representatives to investigate the effects of curing time and temperature on bonding strength. First, according to the indication of TGA results, curing temperatures in the range of 210~270 °C were selected, and 30 min curing time was applied. The results in Figure 4 show that MX almost had no bonding strength when it was cured at 210 °C, whereas MXU exhibited bonding strengths of 10.54 MPa. This is consistent with the fact that X–U condensation is faster than M–X condensation. With the curing temperature elevated to 230 °C, the bonding strength of MX sharply increased to 14.35 MPa while the bonding strength of MXU did not increase obviously. At 250 °C, the highest bonding strength of 19.38 MPa was achieved for MX. A slight decrease at 270 °C may be attributed to the thermal oxidation of the resin since curing was performed in air. Differently, the bonding strength of MXU still fluctuated around 14 MPa, along with the elevated temperature.

To investigate the effect of curing time on bonding strength, curing at 250 °C for a prolonged time was performed. The results in Figure 4b show that 30 min curing for MX led to the highest bonding strength. A more obvious decrease in bonding strength caused by oxidation was observed along with elongated curing time. Interestingly, the bonding strength of MXU increased rapidly with prolonged curing time and reached the highest value of 21.9 MPa at 90 min. A possible reason for this post-cure behavior is that different condensation reactions were involved. At the early stage of cure, X–U and U–U condensations occurred between the branched polymers that contain X and U terminals. Due to the post-added U, small linear X–U polymers were also formed during synthesis, and during curing, these polymers condensed to form larger polymers and/or further reacted with branched polymers. Such a process was delayed but also contributed to the formation of the crosslinked network. It cannot be ruled out that a portion of linear polymers remained in cured resin, but they can also contribute to bonding strength as they would have high molecular weight.

With the optimal curing conditions (250 °C, 30 min for MX and 90 min for MXU, respectively), steel adhesion strengths of more resins with different molar ratios were tested, and the results were given in Supplementary Figure S10a,b. For MX, the resin MX-1.5 presented the highest bonding strength. It is unexpected that MXU-144 exhibited obviously higher bonding strength than other resins. As indicated by DSC results, this resin has the lowest T_g_, probably due to a lower crosslinking degree caused by more excessive urea. However, for adhesion property, more urea units may increase the polarity of the polymers and enhance the van der Walls interaction between substrate surfaces. Especially for metal bonding, more oxygen- and nitrogen-containing units can lead to stronger surface coordination.

To further evaluate the adhesion performance of the two resins, bonding strengths for various substrates were measured. The results in Figure 4c show that MX presented 13~19 MPa for metal and ceramic substrates. The highest bonding strength of 27.46 MPa was achieved for carbon fiber-reinforced epoxy composites (CF). In contrast, MXU exhibited higher bonding strength for steel, Cu, and ceramic substrates but relatively lower bonding strength (22.74 MPa) for CF. For bonding CF-involved composite (hybrid) substrates (Figure 4d), MX and MXU presented similar performances, and the values are in the narrower range of 15~21 MPa. The resistance to strong base and acid was also tested by measuring CF bonding strengths after soaking the specimens in 2 mol L^−1^ NaOH and H_2_SO_4_ solution for 72 h, and the results are given in Figure 4e. For MX resin, the bonding strength declined by 26% and 29% in NaOH and H_2_SO_4_, respectively, indicating alkaline acidic and hydrolysis led to partial degradation of the cured network. In contrast, only an 8.9% and 3.4% decline were observed for MXU, suggesting MXU resin is much more stable toward hydrolysis.

**Figure 5:**
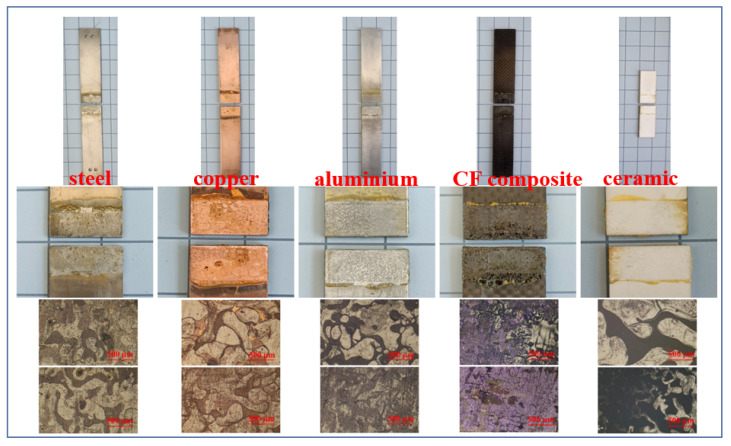
Camera and microscopic images for the tested specimens and failed surfaces for different substrates.

To understand the failure mode, the images for the failed surfaces of the tested specimens (MX bonded) are given in Figure 5. For metal substrates, the metal surface was still covered by adhesive in some sheared areas, but a portion of the metal surface was exposed, indicating the failure mode included both cohesive failure and adhesion failure. The microscopic images for all metal substrates and ceramics show that the cured adhesive has a smooth and arcuate edge, which may be produced by the shrinking of the adhesive during curing. This is also responsible for the exposed area of the substrate surface. For CF composite, substrate failure occurred, which is consistent with the highest bonding strength shown in Figure 4c.

**Figure 6:**
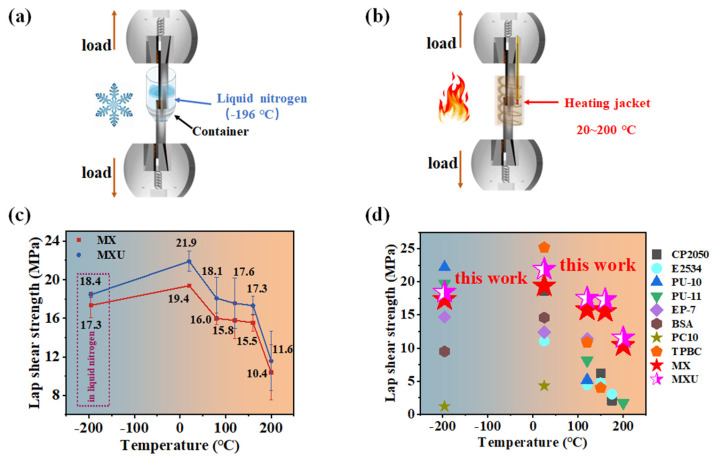
(**a**) Test of steel bonding strength at cryogenic temperature. (**b**) Test of steel bonding strength at elevated temperature. (**c**) Measured steel bonding strengths at different temperatures. (**d**) Comparison of bonding strengths between MX/MXU resins and the structural adhesives in the literature and commercial products (Key: CP2050 [25] and E2534 [26] represent commercial Epoxy-Phenolic and Two-Component Epoxy adhesives, respectively; PU-10 [14], PU-11 [14] represent different polyurethane adhesives; EP-7 [27] represents Bisphenol A epoxy adhesives; BSA [28], PC10 [29] represent supramolecular adhesive; TPBC represents TPBC-toughened epoxy adhesive [12]).

To estimate the resistance of the two resins to extreme temperatures, steel bonding performances under cryogenic (−196 °C) and elevated temperatures were tested using the devices shown in Figure 6a,b. The results in Figure 6c indicate that both MX and MXU are highly resistant to cryogenic temperature, as 84% and 89% of room temperature bonding strengths were retained, respectively. The cryogenic bonding strength of 17~18 MPa is competitive with the performance of commercial polyurethane and epoxy and superior to the recently developed supramolecular adhesives for cryogenic use (Figure 6d). At elevated temperatures of 60~150 °C, the bonding strengths declined but maintained at high values of 15~16 MPa and 17~18 MPa, respectively, for MX and MXU. Such high-temperature performance is much superior to that of previously studied MH and MHU resins (2~5 MPa at 140 °C) [24] and surpasses most of the high-performance commercial and the literature-reported structural resins [14,25,26,27,28,29] (Figure 6d). Note that the good retention of bonding strength in a wide range of 60~150 °C suggests that the decline was not caused by the transition from glass state to elastic state. That is to say, the T_g_ of the two resins should be well above 150 °C. For MX-1.5 resin, the DSC-determined T_g_ ≈ 179 °C support this inference, but for MXU-144, the determined T_g_ ≈ 150 °C may have been underestimated. When the ambient temperature was elevated to 200 °C, a further decline of bonding strength occurred for both resins. However, the bonding strength of 10~11 MPa is still well above the general criterion (>6.9 MPa) for structural use. To the best of our knowledge, so far, only polyimide-based adhesives have been able to operate at temperatures above 200 °C [30].

**Figure 7:**
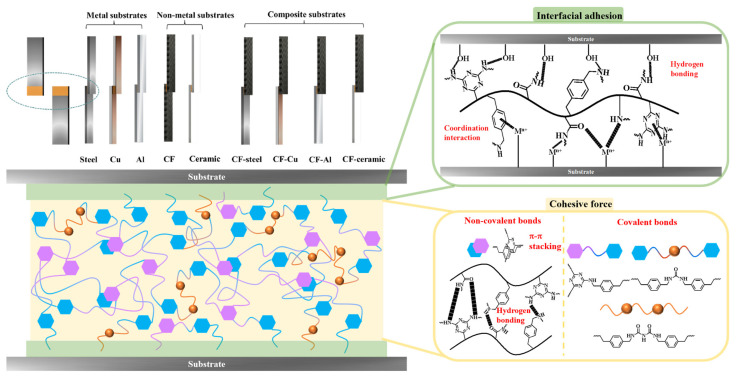
Proposed cured network and adhesion mechanism for different substrate materials.

To rationalize the excellent adhesion property of MX and MXU resins, Figure 7 was drawn to illustrate the proposed adhesion mechanism. The strong, cohesive strength originates from both primary (covalent) and secondary (weak interaction) bonds. After curing, the covalent M–X linkages in MX resin, M–X, X–U, and U–U linkages in MUX are formed and contribute mainly to the cohesive strength. As the resin contains rich aromatic units, the M–M, X–X, and M–X π–π stacking interactions would be very strong and also contribute to the cohesive strength. Additionally, a hydrogen bonding network can also be formed. These long-distance interactions should also be an important factor in the formation of the toughened network in a cryogenic environment. For surface adhesion phenomena, there are several theories that are proposed to explain the interactions between the substrate surface and adhesive, such as mechanical interlocking, absorption (surface wetting), diffusion, electrostatic force, and so on. However, in most cases, they need to be combined to rationalize a specific adhesion system because most of the theories share common and more essential effects on a micro scale; these effects include interfacial covalent bonding and noncovalent bonding such as hydrogen bonding, coordination, and van der Waals interactions, et al. In this work, for metal substrates, we speculate that the coordination effect is one of the important factors as the MX and MXU polymers contain rich oxygen and nitrogen elements, as well as rich aromatic fragments. Particularly, the lone-paired electrons from amino and carbonyl groups, as well as the π electrons from aromatic fragments, can interact strongly with the empty orbitals of metal, namely donor–acceptor (coordination) interactions, leading to strong metal bonding strength. Mechanical interlocking may also contribute to the adhesion strength as the substrate surfaces were sanded before use. Despite the two resins presenting somewhat different bonding strengths, the overall performance indicates that both of them are excellent structural adhesives. In particular, the excellent interface compatibility for different substrates suggests that they are ideal matrices for the fabrication of various composites.

## 4. Conclusions

In conclusion, we successfully developed two high-performance structural adhesives that can work in both high-temperature and cryogenic environments. Particularly, the high bonding strength of 10~17 MPa at 150~200 °C makes the adhesives much more superior to the majority of the commercial and literature-reported organic adhesives. The outstanding high-temperature adhesion property can be attributed to the rich rigid aromatic units incorporated in the polymer structure, which endow the resins with high T_g_. The excellent cryogenic temperature resistance is ascribed to the flexibility of the crosslinked network originating from the dispersed rotatable methylene linkages. The facile and low-cost synthesis makes the resins easy to be commercialized.

## Figures and Tables

**Table 1:** The GPC results for MX and MXU resins.

Molar Ratio	M_n_	M_w_	PDI
M:X = 1:1.5	1.5 × 10^4^	8.3 × 10^4^	5.45
M:X = 1:4	2.1 × 10^4^	9.3 × 10^4^	4.47
M:X:U = 1:4:4	2.4 × 10^4^	7.4 × 10^4^	3.09

## Data Availability

Data sharing is not applicable to this article.

## Supplementary Materials

The following supporting information can be downloaded at: https://www.mdpi.com/article/10.3390/polym15102284/s1, Figure S1: The ^13^C-NMR spectrum for pure X; Figure S2. The ^13^C-NMR spectrum for MX-1.5 resin; Figure S3: The ^13^C-NMR spectrum for MXU-144 resin; Figure S4: The ESI-MS spectrum of the MX-1.5 resin (200~500 Da); Figure S5: The ESI-MS spectrum of the MX-1.5 resin (500~800 Da); Figure S6: The ESI-MS spectrum of the MX-1.5 resin (800~2000 Da); Figure S7: The ESI-MS spectrum of the MXU-144 (200~500 Da); Figure S8: The ESI-MS spectrum of the MXU-144 resin (500~800 Da); Figure S9: The ESI-MS spectrum of the MXU-144 resin (800~2000 Da); Figure S10. (a) Steel bonding strengths of MX resins synthesized with different X:M molar ratios. (b) Steel bonding strengths of MXU resins synthesized with different M:X:U molar ratios.

## References

[1] Kadiyala A.K., Sharma M., Bijwe J. (2016). Exploration of thermoplastic polyimide as high temperature adhesive and understanding the interfacial chemistry using XPS, ToF-SIMS and Raman spectroscopy. Mater. Des..

[2] Liu X.-J., Zheng M.-S., Chen G., Dang Z.-M., Zha J.-W. (2022). High-temperature polyimide dielectric materials for energy storage: Theory, design, preparation and properties. Energy Environ. Sci..

[3] Gleissner C., Landsiedel J., Bechtold T., Pham T. (2022). Surface Activation of High Performance Polymer Fibers: A Review. Polym. Rev..

[4] Li S., Wang H., Liu M., Peng C., Wu Z. (2019). Epoxy-functionalized polysiloxane reinforced epoxy resin for cryogenic application. J. Appl. Polym. Sci..

[5] Sun X., Chen S., Qu B., Zheng Y., Liu X., Li W., Wang R., Chen Q., Zhuo D. (2023). Novel photosensitive resin simultaneously with outstanding cryogenic strength and toughness for digital light processing three-dimensional Printing. Mater. Today Commun..

[6] Acar O., Varis S., Isık T., Tirkes S., Demir M.M. (2018). Synthesis and characterization of novel high temperature structural adhesives based on nadic end capped MDA-BTDA-ODA copolyimide. Mater. Res. Express.

[7] Sargent J.P. (1994). Adherend surface morphology and its influence on the peel strength of adhesive joints bonded with modified phenolic and epoxy structural adhesives. Int. J. Adhes. Adhes..

[8] Ge Y., Zhang X., Dai T., Wang Y., Ling Y., Xu Z., Heng Z., Liang M., Zou H. (2022). Super-Flexibility and High-Temperature Adhesion of Epoxy Structural Adhesives Endowed by Homogeneous Rigid–Flexible Crosslinking Networks. Ind. Eng. Chem. Res..

[9] Kumar P., Patnaik A., Chaudhary S. (2017). A review on application of structural adhesives in concrete and steel–concrete composite and factors influencing the performance of composite connections. Int. J. Adhes. Adhes..

[10] Stefano D., Ingrid C., Olivier D., Laurence C., Andrea T., Valentina C., Monica F. (2020). Adhesive Joining of Zerodur–CFRP–Zerodur Sandwich Structures for Aerospace Applications. Macromol. Mater. Eng..

[11] Galvez P., Abenojar J., Martinez M.A. (2019). Durability of steel CFRP structural adhesive joints with polyurethane adhesives. Compos. Part B Eng..

[12] Zhao L., Li H., Qiao Y., Bai X., Wang D., Qu C., Xiao W., Liu Y., Zhang J. (2022). Accelerated-curing epoxy structural film adhesive for bonding lightweight honeycomb sandwich structures. J. Appl. Polym. Sci..

[13] Yue C., Dong S., Weng L., Wang Y., Zhao L. (2021). Environmental Resistance and Fatigue Behaviors of Epoxy/Nano-Boron Nitride Thermally Conductive Structural Film Adhesive Toughened by Polyphenoxy. Polymers.

[14] Sandler S.R., Berg F.R. (1965). Polyurethanes as cryogenic adhesives. J. Appl. Polym. Sci..

[15] Chen D.S., Ma C.C.M., Hsia H.C., Wang W.N., Lin S.R. (1994). Preparation and characterization of cryogenic adhesives. I. Glycidyl-terminated polyurethane resins. J. Appl. Polym. Sci..

[16] Galvez P., Lopez de Armentia S., Abenojar J., Martinez M.A. (2020). Effect of moisture and temperature on thermal and mechanical properties of structural polyurethane adhesive joints. Compos. Struct..

[17] Wang D., Xiong X., Ren R., Ma X., Han A., Chen P. (2020). Characterization and properties of high-temperature resistant structure adhesive based on novel toughened bismaleimide resins. High Perform. Polym..

[18] Wang K., Yuan X., Zhan M. (2017). Comparison between microwave and thermal curing of a polyimide adhesive end-caped with phenylethynyl groups. Int. J. Adhes. Adhes..

[19] Wang D., Zhao L., Yang H., Yue C., Li H., Xiao W., Liu C., Qu C. (2022). High temperature and toughened bismaleimide structural film adhesive for high performance CFRP bonding over 300 °C. Polym. Degrad. Stab..

[20] Higginson C.J., Malollari K.G., Xu Y., Kelleghan A.V., Ricapito N.G., Messersmith P.B. (2019). Bioinspired Design Provides High-Strength Benzoxazine Structural Adhesives. Angew. Chem. Int. Ed. Engl..

[21] Hergenrother P.M. (2016). The Use, Design, Synthesis, and Properties of High Performance/High Temperature Polymers: An Overview. High Perform. Polym..

[22] Gouzman I., Grossman E., Verker R., Atar N., Bolker A., Eliaz N. (2019). Advances in Polyimide-Based Materials for Space Applications. Adv. Mater..

[23] Iredale R.J., Ward C., Hamerton I. (2017). Modern advances in bismaleimide resin technology: A 21st century perspective on the chemistry of addition polyimides. Prog. Polym. Sci..

[24] Wang S., Shen Y., Du G., Jiang S., Liu S., Niu H., Li L., Qin T., Duan Z., Li T. (2023). Novel Melamine-based Engineering Thermosets: Facile Synthesis, Extraordinary Thermostability, High Strength and Toughness. Chem. Eng. J..

[25] MatWeb Aremco Corr-Paint™ CP2050-XX High Temperature Protective Coating Epoxy-Phenolic. Glass Fiber Filled. https://matweb.com/search/DataSheet.aspx?MatGUID=5dec12e91e0741daba259a3cc21af8e5.

[26] MatWeb Henkel Loctite® Stycast® E 2534 FR CAT 9 Two-Component Epoxy. https://matweb.com/search/DataSheet.aspx?MatGUID=a8ea83984fc446f6bc91a893406228ad.

[27] Sandler S.R., Berg F.R. (1965). Effect of polarity of bisphenol a epoxy resins on adhesion at cryogenic and elevated temperatures. J. Appl. Polym. Sci..

[28] Sun P., Mei S., Xu J.F., Zhang X. (2022). A Bio-Based Supramolecular Adhesive: Ultra-High Adhesion Strengths at both Ambient and Cryogenic Temperatures and Excellent Multi-Reusability. Adv. Sci..

[29] Li X., Lai J., Deng Y., Song J., Zhao G., Dong S. (2020). Supramolecular Adhesion at Extremely Low Temperatures: A Combined Experimental and Theoretical Investigation. J. Am. Chem. Soc..

[30] Zharinov M.A., Petrova A.P., Babchuk I.V., Akhmadieva K.R. (2022). Thermally Stable Polyimide Structural Adhesives. Polym. Sci. Ser. D.

