# Supplementary material for: Tough Structural Adhesives with Ultra-Resistance to Both High and Cryogenic Temperature
Source: Polymers (Basel). 2023 May 12;15(10):2284. doi: 10.3390/polym15102284 (PMC10223711; doi:10.3390/polym15102284)
Supplement: Supplementary file 1 [file polymers-15-02284-s001.zip › polymers-2388989-supplementary.pdf]

Supplementary Materials

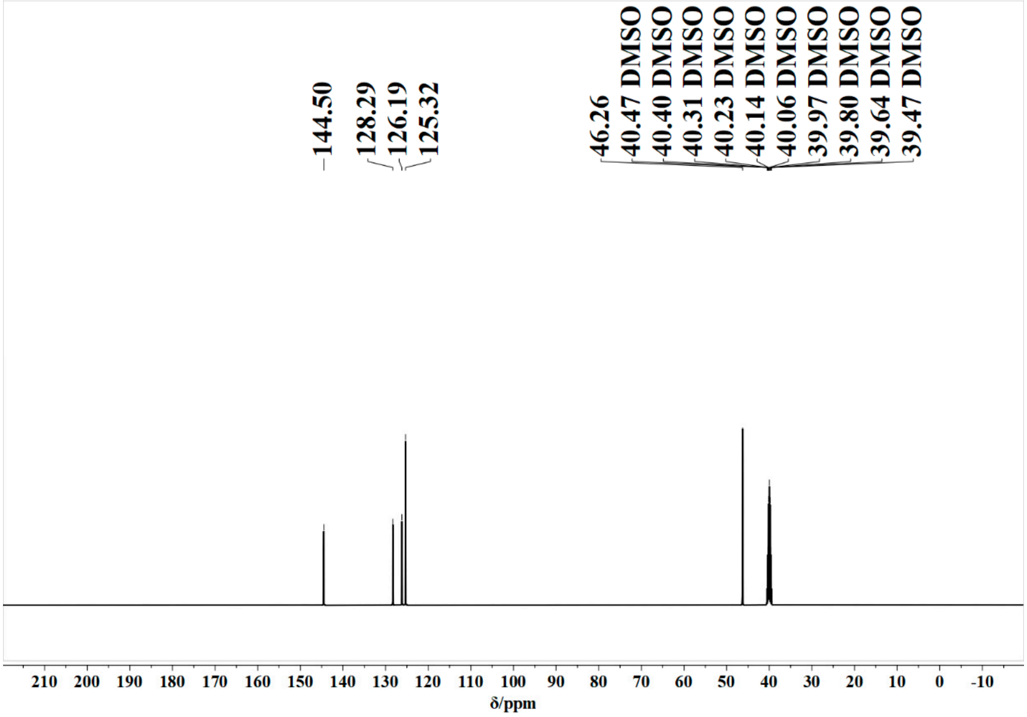

Figure S1. The ^{13}C-NMR spectrum for pure X.

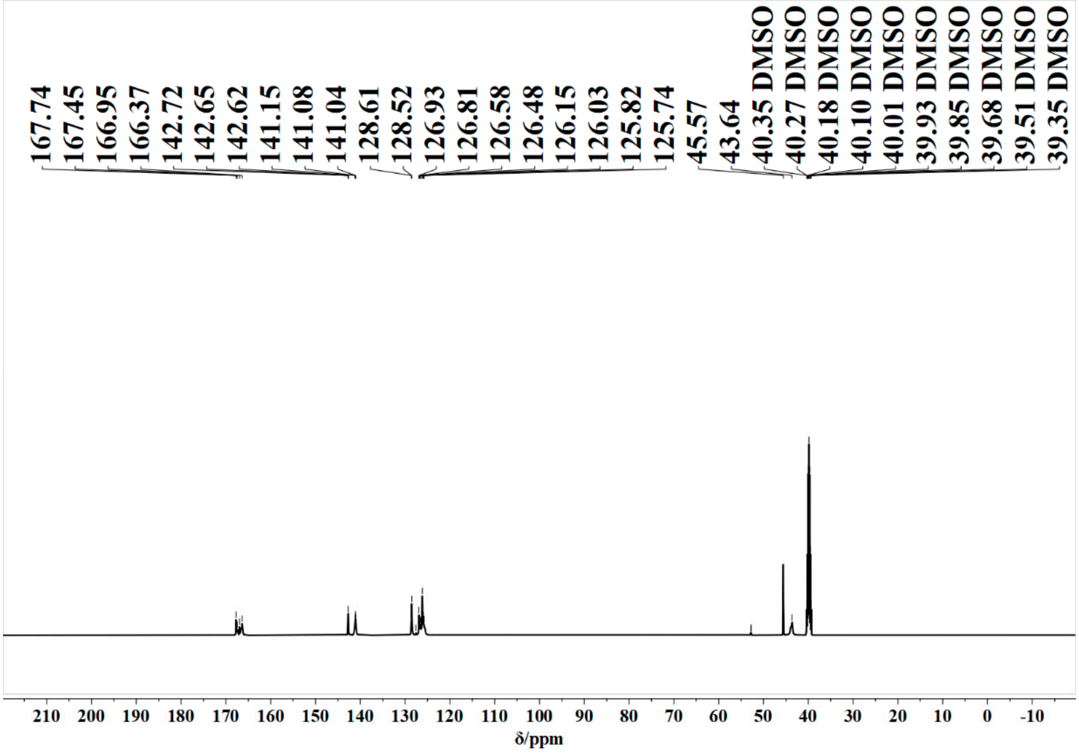

Figure S2. The ^{13}C-NMR spectrum for MX-1.5 resin.

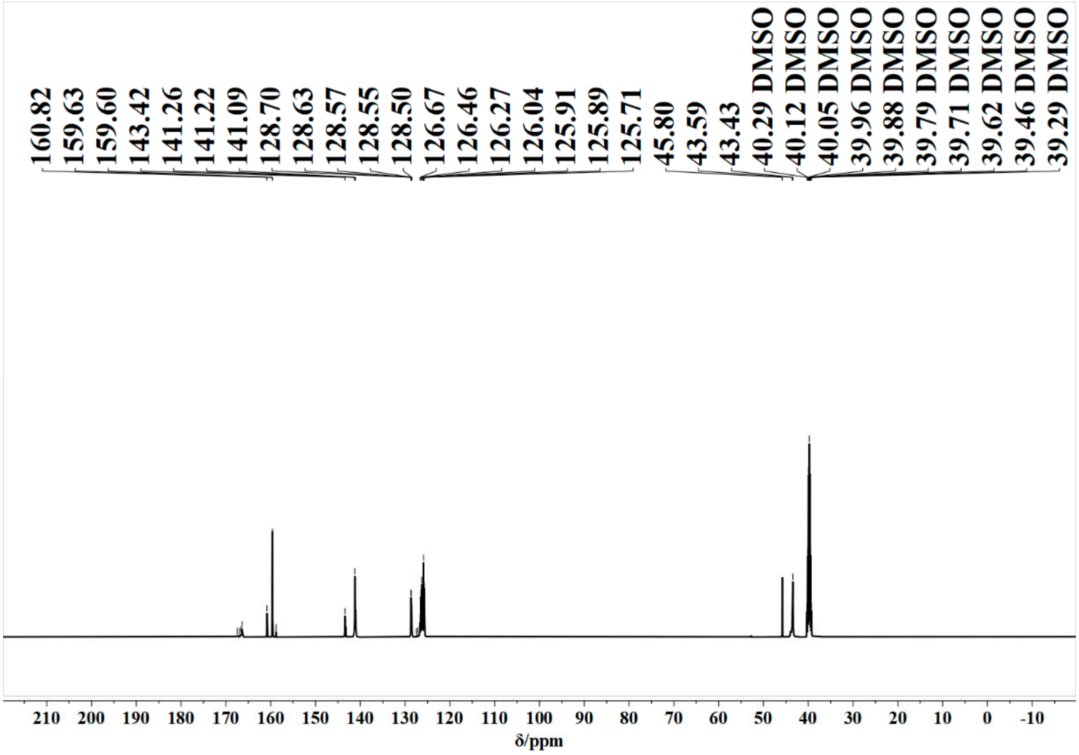

Figure S3. The ^{13}C-NMR spectrum for MXU-144 resin.

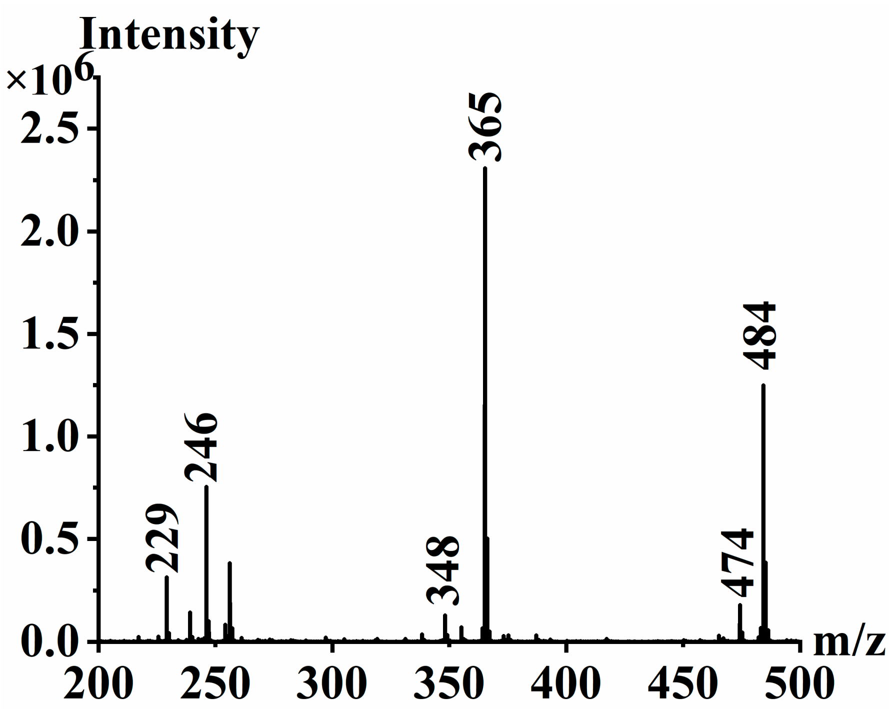

Figure S4. The ESI-MS spectrum of the MX-1.5 resin (200 ~ 500 Da).

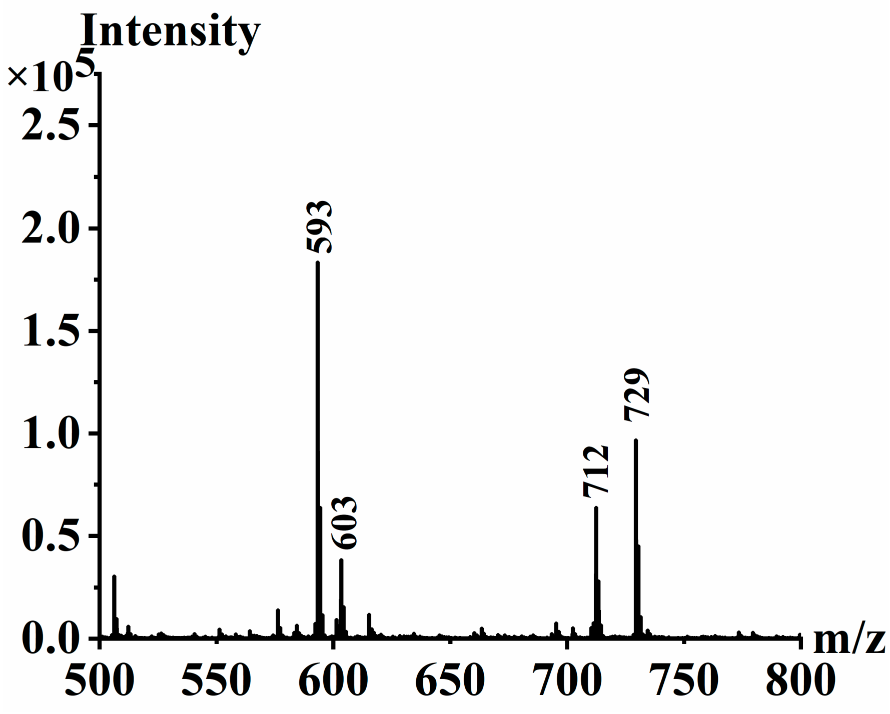

Figure S5. The ESI-MS spectrum of the MX-1.5 resin (500 ~ 800 Da).

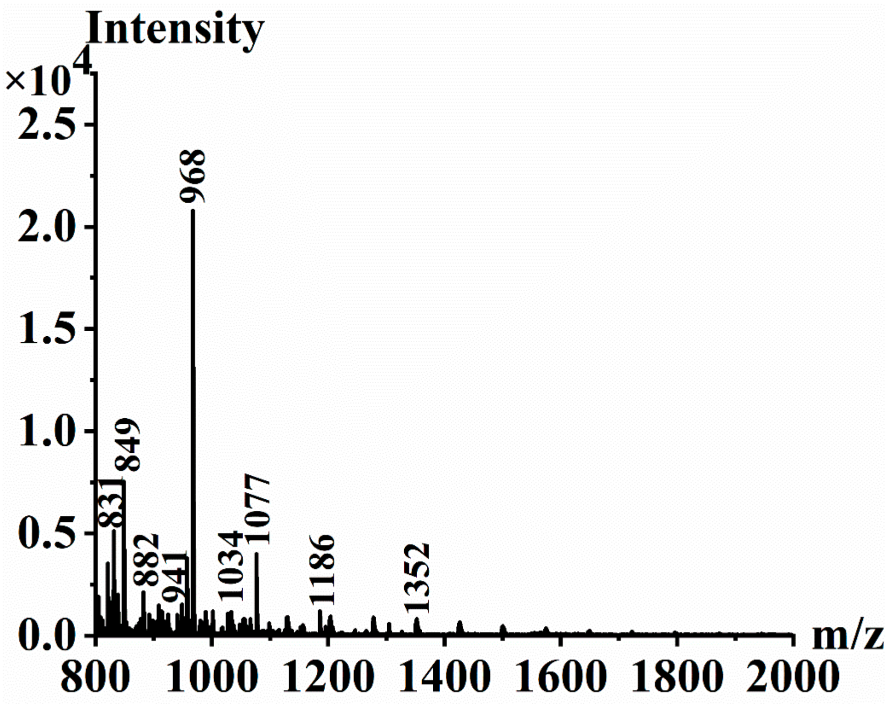

Figure S6. The ESI-MS spectrum of the MX-1.5 resin (800 ~ 2000 Da).

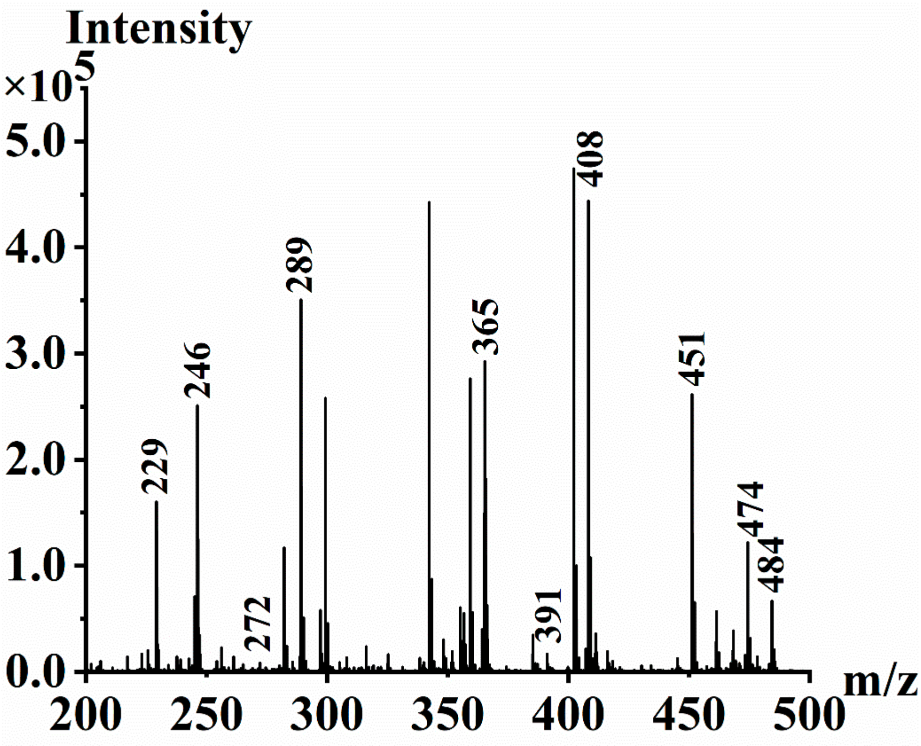

Figure S7. The ESI-MS spectrum of the MXU-144 (200 ~ 500 Da).

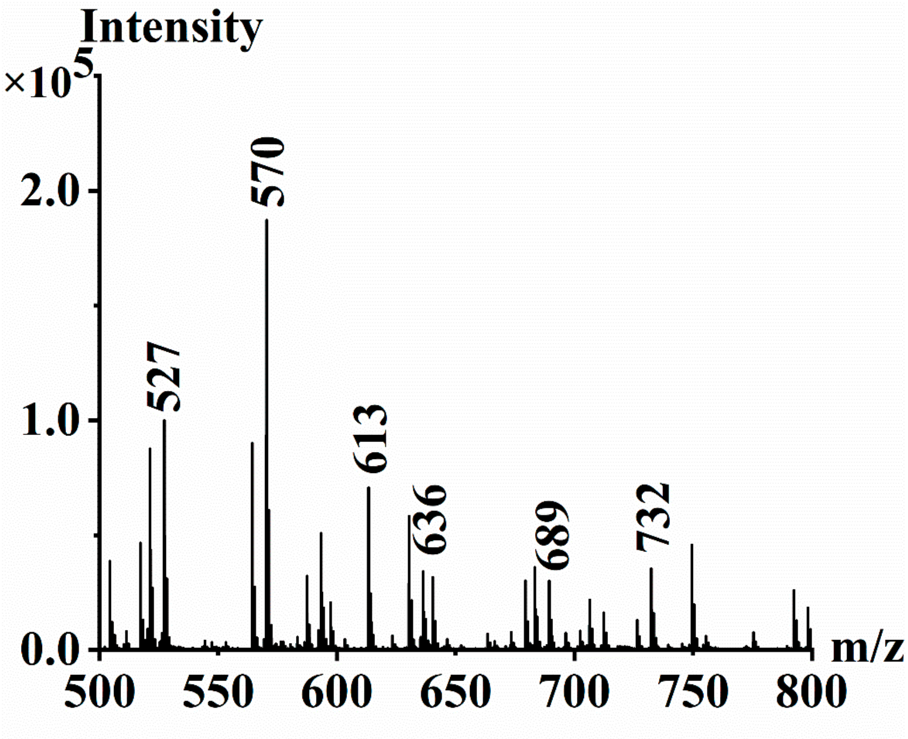

Figure S8. The ESI-MS spectrum of the MXU-144 resin (500 ~ 800 Da).

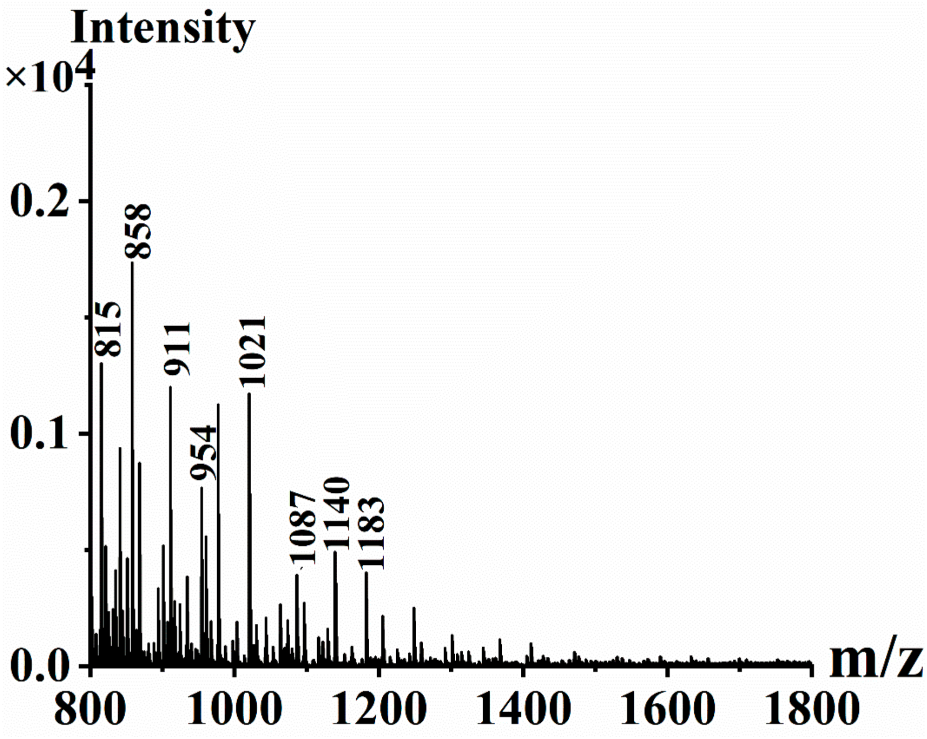

Figure S9. The ESI-MS spectrum of the MXU-144 resin (800 ~ 2000 Da).

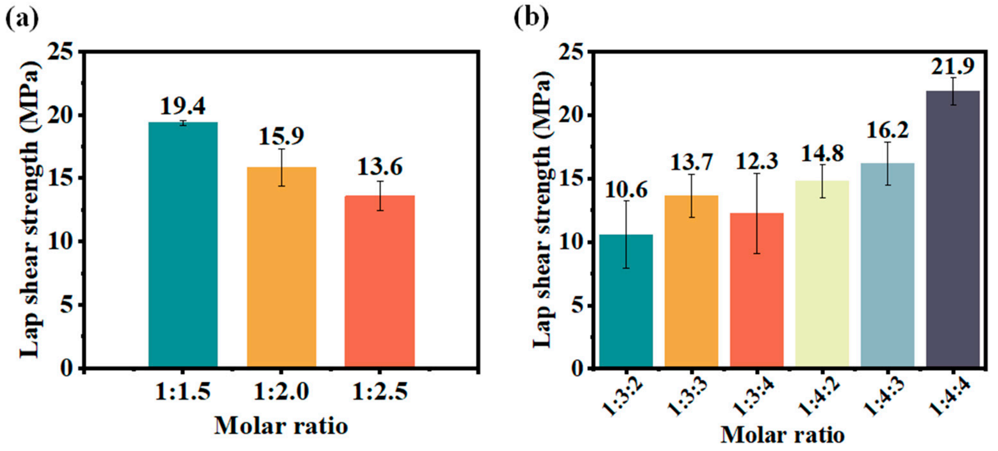

Figure S10. (a) Steel bonding strengths of MX resins synthesized with different X:M molar ratios. (b) Steel bonding strengths of MXU resins synthesized with different M:X:U molar ratios.
